# Genetics and Sex in the Pathogenesis of Amyotrophic Lateral Sclerosis (ALS): Is There a Link?

**DOI:** 10.3390/ijms21103647

**Published:** 2020-05-21

**Authors:** Francesca Trojsi, Giulia D’Alvano, Simona Bonavita, Gioacchino Tedeschi

**Affiliations:** Department of Advanced Medical and Surgical Sciences; MRI Research Center SUN-FISM, Università degli Studi della Campania “Luigi Vanvitelli”, 80138 Naples, Italy; dalvanogiulia@hotmail.it (G.D.); simona.bonavita@unicampania.it (S.B.); gioacchino.tedeschi@unicampania.it (G.T.)

**Keywords:** amyotrophic lateral sclerosis, sex, gender, genetics, *SOD1* mutations, *C9orf72* repeat expansion

## Abstract

Amyotrophic lateral sclerosis (ALS) is a fatal neurodegenerative disease with no known cure. Approximately 90% of ALS cases are sporadic, although multiple genetic risk factors have been recently revealed also in sporadic ALS (SALS). The pathological expansion of a hexanucleotide repeat in *chromosome 9 open reading frame 72* (*C9orf72)* is the most common genetic mutation identified in familial ALS, detected also in 5–10% of SALS patients. *C9orf72*-related ALS phenotype appears to be dependent on several modifiers, including demographic factors. Sex has been reported as an independent factor influencing ALS development, with men found to be more susceptible than women. Exposure to both female and male sex hormones have been shown to influence disease risk or progression. Moreover, interplay between genetics and sex has been widely investigated in ALS preclinical models and in large populations of ALS patients carrying *C9orf72* repeat expansion. In light of the current need for reclassifying ALS patients into pathologically homogenous subgroups potentially responsive to targeted personalized therapies, we aimed to review the recent literature on the role of genetics and sex as both independent and synergic factors, in the pathophysiology, clinical presentation, and prognosis of ALS. Sex-dependent outcomes may lead to optimizing clinical trials for developing patient-specific therapies for ALS.

## 1. Amyotrophic Lateral Sclerosis (ALS): A Multifactorial Neurodegenerative Disease

Amyotrophic Lateral Sclerosis (ALS), also known as Lou Gehrig’s disease, is a fatal neurodegenerative disease characterized by varying phenotypic manifestations due to degeneration of both upper and lower motor neurons [1]. Clinical symptoms include muscle weakness, spasticity, dysphagia, dysarthria, and respiratory failure; up to 50% of the patients develop cognitive and/or behavioral impairment during the disease course, and about 13% present with concomitant behavioral variant frontotemporal dementia (bv-FTD) [1].

Although our understanding of ALS is improving, the pathological pathways that lead to the disease and affect the overall integrity of brain networks are still incompletely understood. Extensive literature provides convincing evidence for a number of cellular and molecular processes found to be implicated in ALS pathogenesis, including, among the most largely studied, excitotoxicity, oxidative stress, impaired protein degradation (proteasome and/or autophagy), toxic protein aggregation, mitochondrial dysfunction, axonal transport, prion-like spreading, dysfunction of non-neuronal cells, neuroinflammation, defects in RNA metabolism, and RNA toxicity [2]. However, defects in some of these pathways may represent a secondary phenomenon, and taking into account that 5–15% of ALS patients have a familial disease (FALS) and that multiple genetic risk factors have been recently revealed for sporadic ALS (SALS) [3], genetics has provided relevant insights regarding pathophysiology underlying ALS. In particular, it is considered a multifactorial disease, influenced by genetic and environmental factors.

Two twin studies, one population- and one clinic-based, have suggested that heritability contributes about 60% to the risk of developing ALS and the unshared environmental component approximately 40% [4], although an analysis applying the genome-wide complex trait analysis algorithm to three genome-wide association studies (GWAS) datasets estimated the overall heritability of ALS to be approximately 21% [5]. More recently, a population-based study by Chiò et al. [6] demonstrated that the spatial–temporal combination of motor and cognitive events leading to the onset and progression of ALS is influenced by demographic (mainly age and sex) and genetic factors. Remarkably, data from ALS population registers in Italy and the Republic of Ireland confirmed that deficits in multiple pathways, reflecting a “multistep” model of disease consistent with a six-step process, are required to develop ALS, although they revealed that a reduced number of steps are detected in patients with ALS with genetic mutations compared to those without mutations [7]. Therefore, these findings from the interrogation of population-based registers further emphasize the need to study the interaction between genetic, demographic, environmental, and lifestyle risk factors.

Among demographic factors, sex has been reported as an independent factor influencing the development of ALS, with men reported to be 2-3 times more susceptible than women [8], especially in case of flail limbs and respiratory phenotypes, with a trend toward a higher frequency in older age [6]. Moreover, the role of sex in clinical presentation and prognosis of ALS patients carrying *superoxide dismutase-1* (*SOD1)* mutations and *chromosome 9 open reading frame 72 (C9orf72)* repeat expansion has also been explored [6,7,8,9,10].

With this background, in the light of the current need to reclassify ALS patients into pathologically homogenous subgroups potentially responsive to targeted personalized therapies, we aimed to review the recent literature on the role of genetics and sex, as both independent and synergic factors, on the pathophysiology, clinical presentation, and prognosis of ALS. With regard to the search strategy and the selection criteria adopted, we searched Pubmed (2010 to April 2020) using the search terms “amyotrophic lateral sclerosis” or “motor neuron disease” in combination with “epidemiology”, “genetics”, “sex”, and “gender”. Further articles were included from reference lists and review articles. The final reference list was generated on the basis of originality and relevance to the topics covered in this manuscript. Emphasis was placed on publications from the last 5 years but did not exclude highly regarded older publications. Table 1 summarizes the most relevant articles among the reviewed and referenced ones, which addressed the interplay between sex and genetics in the ALS pathogenesis.

## 2. The Role of Genetics in ALS Pathogenesis

An increasing number of genetic variants are recognized as associated with ALS [3,11,12]. In 60–80% of patients with FALS, gene mutations of large effect, such as repeat expansions in *C9orf72* (40%), mutations of *SOD1* (20%), *fused in sarcoma (FUS)* (1–5%), and *transactive response DNA binding protein* (*TARBDP)* (1–5%), can be most commonly identified [10,11]. In recent years, advances in massive parallel sequencing approaches such as whole-genome sequencing (WGS) and whole-exome sequencing (WES) [12,13,14], derived from large-scale collaborations which have previously led to GWAS in ALS [15,16,17] and to the international WGS project MINE (www.ProjectMine.com) [18], shed more light on the genetic architecture of ALS. In particular, the latest WGS and WES studies underlined that in ALS, one or a few large-effect, relatively rare variants in each patient seem to contribute most to the disease risk, which is different from conditions such as Alzheimer disease or schizophrenia, in which multiple small-effect variants add up to create risk [12,19,20], suggesting an oligogenic model in ALS [21]. Moreover, this evidence is consistent with the observation of multiple ALS-associated genes co-segregating in some kindreds [21]. Another relevant aspect is the incomplete penetrance in many ALS pedigrees, causing unreliable heritability, especially in the case of reduction of family sizes (i.e., “apparently” SALS) [22], and shown to be age- and sex-dependent in patients carrying *C9orf72* repeat expansion [23]. Of note, ALS-associated genes may be pleiotropic (e.g., linked to several phenotypes), such as for *C9orf72* repeat expansions [24] or other genetic variants [25,26].

**Figure 1:**
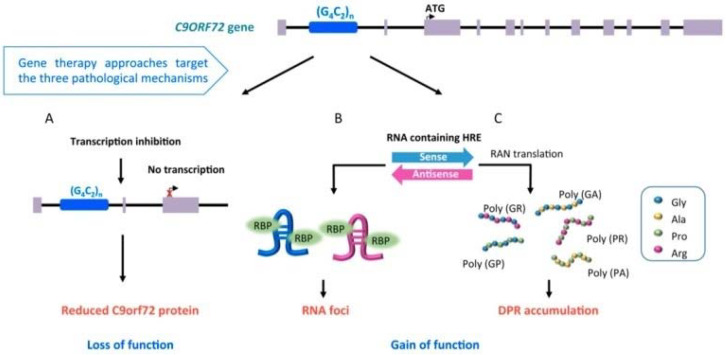
Pathological mechanisms involved in *C9orf72*-ALS: (**A**) transcription inhibition (loss of function); (**B**) detection of RNA foci, involved in impairment of RNA metabolism (gain of function); (**C**) accumulation of toxic dipeptide repeat proteins (DRP) aggregates (gain of function) (derived from Cappella et al. [34], open access article distributed under the terms and conditions of the Creative Commons Attribution (CC BY) license (http://creativecommons.org/licenses/by/4.0/)).

The pathological expansion of a hexanucleotide repeat in *C9orf72* is the most common genetic mutation identified in European and Caucasian patients with ALS, reported in about 40% of patients with FALS, 25% of patients with familial FTD and 5–10% of patients with SALS [16,17]. *C9orf72* expansion influences clinical presentation, with patients carrying the expansion exhibiting higher prevalence of bulbar onset, earlier age at onset, and reduced survival, with higher incidence of comorbid FTD and/or family history of dementia or ALS [27,28,29,30,31,32,33]. Moreover, the detection of repeat expansion in *C9orf72* has been also associated with Parkinsonism, Huntington phenocopies, Alzheimer’s disease, corticobasal degeneration, and psychosis [24]. Although the function of the protein encoded by *C9orf72* is still unknown, there are several theories regarding how the *C9orf72* repeat expansion leads to ALS and FTD [34] (Figure 1): i) loss of C9orf72 protein expression could inhibit autophagy and promote neuroinflammation (Figure 1A) [35,36]; ii) expanded sense and antisense RNA, derived from the expanded gene, form toxic RNA foci that sequester RNA binding proteins, impairing RNA metabolism (Figure 1B) [15,36]; iii) non-canonical repeat-associated non-ATG (RAN) translation of toxic dipeptide repeat proteins aggregates (Figure 1C) [37]. Disease severity and phenotype appear to be dependent on the size of the repeat expansion (which may vary between cell types within an individual (mosaicism)), methylation level at CpG sites [38] and the co-occurrence of genetic variation in other genes (e.g., *transmembrane protein 106B -TMEM106b*, *ataxin-2*- *ATXN2, C6orf10/LOC101929163/HLA-DRB1* pathway, and others) [24,39,40].

Mutations in *copper/zinc SOD1* gene were identified as causative of ALS in 1993 [41] and account for 12–23.5% of FALS and about 7.3% of apparent SALS in Caucasian and European populations [42]. Among Asian populations, in Chinese ALS patients, in which *C9orf72* repeat expansion account for 0–1.5% of SALS and 5.98% of FALS, *SOD1* gene has been revealed as the most common causative gene, accounting for 1.45% of SALS and 25.33% of FALS [43]. Currently, the best-studied mechanism for ALS involves SOD1 [44], and more than 180 missense mutations have been reported in *SOD1* (http://alsod.iop.kcl.ac.uk) to date. The reported heterogeneity in the age of onset and phenotype in *SOD1*-mutated patients [45] is due to different types of mutations that result in a gain of toxicity rather than a loss of enzymatic activity [46] and may be influenced by epigenetic factors such as sex, modifier genes, or environmental factors [47]. With regard to the contribution of rare genetic variants in regulating phenotypic variability, in a WGS analysis on *SOD1*-mutated patients and in a WES analysis on SALS patients, Pang et al. [48] observed that *SOD1*-mutated patients with additional rare variants had shorter survival and that the cumulative effect of rare genetic variants was significant on reducing survival probability in both FALS and SALS.

Mutations in the *TARDBP* gene, encoding the 43 kDa transactive response DNA-binding protein (TDP-43), are described in 1–5% of FALS [49]. Interestingly, phosphorylated TDP-43 (pTDP-43)-positive inclusion bodies have been detected in ubiquitin-positive, tau-negative ALS and FTD cytoplasmatic aggregates, representing the pathological hallmark of the ALS–FTD continuum of neurodegeneration [50,51]. pTDP-43 is, therefore, thought to have an essential role in ALS pathogenesis, suggesting its involvement in both loss-of-function (i.e., perturbation of nuclear TDP-43 function) [52] and gain-of-function (i.e., splicing of selected RNA targets; proteasome inhibition) [53] pathological mechanisms. Moreover, progression of ALS pathology can be divided into four stages on the basis of changes in the brain and spine distribution of intraneuronal pTDP-43 aggregates [51,54]. Mutations in the gene *FUS*, encoding a second RNA/DNA-binding protein, have been associated with 1–5% of FALS. Similarly to the mechanisms associated with pTDP-43 pathology, FUS toxicity in ALS has been related to dysfunction in RNA processing and in maintaining the DNA damage response [49,55].

More recently, identification of several other genes carrying rare causal variants, including *valosin-containing protein (VCP)*, *sequestosome 1 (SQTSM1)*, *ubiquilin 2 (UBQLN2)*, *TANK-binding kinase 1 (TBK1)*, *optineurin (OPTN)*, *coiled-coil-helix-coiled-coil-helix domain-containing 10 (CHCHD10)*, *tubulin alpha 4A (TUBA4A)*, *matrin 3 (MATR3)*, *cyclin F (CCNF)*, *never in mitosis*, *gene A -related kinase 1 (NEK1)*, *chromosome 21 open reading frame 2 (C21orf2)*, *Annexin A11 (ANXA11)*, *T cell-restricted intracellular antigen-1 (TIA1)*, *and glycosyltransferase 8 domain containing 1 (GLT8D1)*, has contributed to shedding more light on the genetic architecture of ALS and FTD and the related molecular pathways [12,56,57]. In particular, among the most recently described mutations in the ALS–FTD spectrum of neurodegeneration, *TBK1* loss-of-function mutations, producing premature termination codons that cause nonsense-mediated mRNA decay and resulting in the loss of mutant transcript and subsequent loss or reduction of *TBK1* protein, has been associated with both ALS and FTD [58,59]. From a phenotypic point of view, a genotype–phenotype analysis of a Belgian population shows that *TBK1* mutated carriers may present ALS and/or FTD with a relatively late age at onset or extrapyramidal symptoms, and associated memory deficits [60]. Of note, a WGS analysis performed on pathologically confirmed Frontotemporal Lobar Degeneration-TDP-43 patients, negative for *C9orf72* and *GRN* mutations, revealed a heterozygous missense mutation in *TBK1* in three patients and a loss-of-function mutation in *TBK1* associated with deletion of exons 13–15 of *OPTN* [61], thereby corroborating the idea of an oligogenic model of disease in the ALS–FTD spectrum caused by multiple rare variants with additive or synergistic effects on disease presentation (i.e., both *TBK1* and *OPTN* are involved in the autophagy pathways) [12]. Rare variants in *NEK1* were identified in 3–5% of ALS populations [13,58,62,63,64]. With regard to the pathogenic pathways involving NEK proteins, which belong to the protein family of NIMA-related serine/threonine kinases, they participate in maintaining the cytoskeleton network [65] together with TUBA4A [66] and profilin 1 [67]. Moreover, *NEK1* interacts with the chromosome 21 open reading frame 2 (C21orf2) in DNA damage repair, together with vesicle-associated membrane-protein-associated protein B/C (VAPB) and alsin (ALS2) [13,68]. Remarkably, *C21orf2* was also recently identified as an ALS-associated gene [14], and autosomal recessive mutations in both *NEK1* and *C21orf2* are linked to a skeletal disorder, axial spondylometaphyseal dysplasia [69,70], further highlighting the genetic and functional link between these genes and proteins. Of note, in ALS and ALS–FTD patients, additional mutations in ALS genes may be revealed in *NEK1* carriers, as identified in two Belgian siblings with FALS and cognitive impairment in which *NEK1* p.Ser1036* loss-of-function mutation was associated with *C9orf72* repeat expansion and *TUBA4A* p.Thr381Met variant, presenting with early disease onsets [63,71]. More recently, mutations in *GLT8D1*, encoding a widely expressed glycosyltransferase enzyme, has been associated with FALS [57], resulting in higher substrate affinity of the mutated enzyme and in a dominant-negative effect with a competitive antagonism of wild-type enzyme function [57].

With this multifaceted genetic background and bearing in mind also the recently proposed “multistep” model of disease in ALS consistent with a six-step process, in the patients carrying genetic mutations associated with ALS, the number of steps necessary to start the neurodegenerative process has been shown to be reduced compared to cases without mutation (i.e., lower for *SOD1*, intermediate for *C9orf72*, and higher for *TARDBP)* [7]. These findings provide evidence for the investigation of demographic, environmental, and lifestyle risk factors that could interact with genetics, especially in individuals with identified mutations rather than in those apparently with SALS, because such additional factors will be fewer in number per person in the former ones and likely of larger effect size [7]. Among non-genetic triggers of pathological steps, the role of sex has been also investigated and debated [72].

## 3. The Role of Sex in ALS Pathogenesis

ALS is characterized by a sexual dimorphism, the most obvious being the higher risk of developing the disease in men [8]. However, a recent population-based study on prospective data from the Piemonte and Valle d’Aosta (in Northern Italy) ALS register has revealed that the age-adjusted incidence rate of ALS increased from 1994 to 2014 and the adjusted rate ratio of men to women decreased from 1.27:1 (1995–2004) to 1.17:1 (2005–2014) [73], probably due to the effect of the modified lifestyle in women across time, which may have caused a larger exposure of women to exogenous risk factors (e.g., cigarette smoking, engagement in manual work) [73]. Importantly, an epidemiologic analysis on demographic, clinical, and lifestyle data from 2008 to 2012 derived from European population-based ALS registries (i.e., from France, Ireland, Italy, United Kingdom, and Serbia) revealed an inverse correlation between the odds of having ALS and sport-related physical activity in women but not in men [74]. However, this different association between ALS and sport-related physical activity in the two sexes has been shown to be less evident after menopause [75], thereby suggesting the potential influence of female hormones, in particular estrogen and progesterone, as protective factors against ALS triggers. In a population-based, case-control study in the Netherlands between 2006 and 2009, de Jong et al. [76] demonstrated that longer reproductive time-span and lifetime endogenous estrogen exposure were associated with a longer survival in ALS patients. Conversely, contrasting results are available on the role of exposure to exogenous estrogens and progestogens as risk factors for ALS, with some studies reporting a decreased risk in women treated with oral contraceptives [77] and others reporting no association between postmenopausal hormone use and development of ALS [78]. Moreover, among sex hormone drugs, in a population-based case-control study of 10,450 U.S. Medicare participants, tamoxifen was related to lower ALS risk and testosterone to a higher risk in women [79].

Evidence from ALS preclinical models have been useful in demonstrating the protective effects of female hormones, mainly attributed to their direct influence on neural and muscular cells [80], preventing cell death, and to a reduction of neuroinflammation, among the main pathogenic mechanisms related to ALS [81]. In particular, a study performed on SOD1 G93A mice revealed that progesterone slowed down the progression of the disease and extended the life span of the affected male mice, without delaying the symptom onset [80]. Progesterone might delay the neurodegeneration process by activating autophagy degradation of mutant *SOD1*. On the other hand, Heitzer et al. [81] revealed that in male SOD1 G93A ALS mice, the treatment with 17β-Estradiol increases the survival of motoneurons, suggesting that this could be related to the downregulation of several components of the inflammatory response (e.g., NLRP3, IL1beta, and activated caspase 1) abnormally elevated in the spinal cord of the affected mice [81].

With regard to the effects of male sex hormones on the ALS risk, it has been hypothesized that androgens might affect motoneurons organization and that prenatal testosterone, irrespective of gender, might play a role in the development of ALS later in life [82]. Considering that the ratio between the lengths of the index finger and the ring finger (2D:4D ratio) may help to estimate the exposure to testosterone in utero for both males and female, with a reduced ratio associating with increased testosterone, Vivekananda et al. [82] demonstrated that, independently of sex, in ALS patients, the 2D:4D ratio was lower than in controls. This finding suggested that increased prenatal testosterone levels could be an independent risk factor for ALS [82]. Moreover, Herron et al. [83] revealed that, in the SOD1 G93A mice model, only males presented enlarged C-boutons, a specific class of synaptic input that originates from a small cluster of spinal interneurons: perturbations in their inputs to motoneurons may contribute to altered excitability and degeneration of motoneurons. In particular, testosterone-mediated effects on cholinergic C-boutons might lead to their selective perturbation in male SOD1 G93A mice [83], since testosterone has been shown to influence the number and size of synapses on motoneurons [84]. Furthermore, the potential role played by circulating androgens in the pathogenesis of ALS may also be suggested by the absence of androgen receptors in neurons spared by ALS (cranial nerves III, IV, and VI) [85]. With regard to the investigations on the correlation between androgens exposure and ALS risk in humans, Gargiulo-Monachelli et al. [86] showed that female ALS patients had an increased level of circulating testosterone compared to healthy females and that the circulating levels of testosterone, dehydroepiandrosterone (and its sulfate), and progesterone were elevated with age in the patient group, differently from healthy females, in whom the three hormones significantly declined across time. In addition, in ALS patients who showed higher testosterone levels and a lower progesterone/free testosterone ratio, a faster worsening of respiratory parameters was observed [86]. In line with these findings, Pfeiffer et al. [79] have described in a cohort of U.S. Medicare beneficiaries an association between testosterone use and elevated ALS risk, particularly in women.

Large population-based studies on data from the Piemonte and Valle d’Aosta register [6,87] revealed an association between sex and motor and cognitive phenotypes in ALS. Male sex was found to be associated with flail arm and respiratory phenotypes, and female sex with bulbar phenotype, but only through an interaction with age [6,87]. With regard to cognitive phenotypes, in the ALS–FTD subgroup, women were found to be more affected than men with increasing age [6]. In agreement with this evidence of an increased vulnerability in cognitive functions in ALS females, Palmieri et al. [88] revealed a greater executive impairment in ALS females than in ALS males. Conversely, Flaherty et al. [89] reported a protective role of estrogens against Frontotemporal Lobar Degeneration, resulting in a preservation of executive abilities in ALS females.

Interestingly, with regard to the investigations on the potential correlation between sex and disease duration/survival in ALS, Pupillo et al. [90] performed a population-based study on data from the Lombardia ALS register, collected from 1998 to 2002, addressing the role of sex, among other demographic and clinical variables, as a predictor of survival: longer survival was predicted by the male gender, considering that standardized mortality ratios at 10 years, used to assess the 10-year excess mortality of ALS patients, were found to be higher until age 75 year in comparison to the general population, predominately in women, and became non-significant for males thereafter. Of note, the Dutch case-control study by de Jong et al. [76] revealed that, among the ALS females, a longer survival was described only in those with more protracted reproductive time-span, exposed longer to endogenous estrogens. However, although largely explored, data about the potential association between sex and risk of developing ALS in humans are still scattered, needing the assessment of co-occurrent risk factors, such as the potential interplay with genetics.

### Key Points

#### Question: Are the Female and/or the Male Hormones Associated with ALS Development and Prognosis?

Findings in favor of the neuroprotective role of the female hormones, differently from the male hormones [74,75,76,77,79,80,81,82,83,84,85,86,89,90]

There is an inverse correlation between the odds of having ALS and sport-related physical activity in women but not in men, and this correlation becomes less evident after menopause. Longer reproductive time-span and lifetime endogenous estrogen exposure are associated with a longer survival in ALS patients. A decreased risk of developing ALS is reported also in women treated with oral contraceptives. Furthermore, tamoxifen use is related to lower ALS risk, and testosterone use to a higher risk in women. Higher testosterone levels and lower progesterone/free testosterone ratio are associated with a faster worsening of respiratory parameters.

With regard to cognitive phenotypes, estrogens are protective against Frontotemporal Lobar Degeneration.

Preclinical models (i.e., SOD1 G93A mice or rats) support the protective role of progesterone and 17β-estradiol, mainly attributed to their direct effect on neural and muscular cells, preventing cell death, and to a reduction of neuroinflammation. Conversely, androgens seem to affect motoneurons organization, and prenatal exposure to testosterone, irrespective of gender, might play a role in the development of ALS later in life. Finally, neurons spared by ALS (cranial nerves III, IV, and VI) do not show androgen receptors.

Findings contrasting with the neuroprotective role of the female hormones [6,73,78,88].

The crude incidence rate of ALS increased by 9% during the 1995 to 2014 period, after age and sex adjustment and mostly limited to women. This increase can be partly ascribed to the effect of the modified lifestyle in women across time, which may have caused a larger exposure of women to exogenous risk factors. No association is described between postmenopausal hormone use and the development of ALS.

With regard to cognitive phenotypes, among ALS–FTD patients, women may become more affected than men at increasing age. Moreover, ALS females may exhibit a greater executive impairment than males.

## 4. Interplay between Sex and Genetics in ALS Pathogenesis

Several studies have addressed the hypothesis that sex may influence ALS triggers due to genetic mutations in ALS preclinical models and in humans. In particular, *SOD1* mutations seem to have a sex-specific effect on the proliferation and differentiation rate of motoneuron cells, according to the study performed in neural progenitor cells (rNPCs) from SOD1 G93A transgenic rats [91]. Li et al. [91] showed that in male rNPCs, but not in female rNPCs, the overexpression of mutant *SOD1* decreased significantly their proliferative and differentiating potential, and sensitivity to oxidative stress was increased, thereby suggesting that sexual dimorphism in ALS can be attributed to intrinsic cellular differences of sex and SOD1 G93A transgene, more than to gonadal steroids. Accordingly, Hayes-Punzo et al. [92] revealed that in SOD1 G93A transgenic, gonadectomy had no significant effect on disease onset or progression, corroborating the idea that gonadal steroids are not key modulators in ALS. On the other hand, a more recent study reported that ovariectomy resulted in earlier disease onset and attenuated anti-inflammatory and anti-apoptotic actions of the estrogens in SOD1 G39A transgenic mice, as a consequence of downregulation of aromatase and estrogen receptor α expression and inhibition of anti-inflammatory and anti-apoptotic factors (e.g., arginase 1, transforming growth factor beta -TGFβ, B-cell lymphoma 2 -Bcl 2) [93].

Another hypothesis regards the role of allelic variants of Chromogranin B (CHGB), a component of secretory vesicles that acts as a chaperone-like protein, in exacerbating sexual differences in the presence of *SOD1* mutations [94]. Ohta et al. [94] found that overexpression of the allelic variant CHGBL413 transgene in SOD1 G37R mice precipitated disease onset and increased its duration specifically in female mice, likely by restraining the secretion of misfolded *SOD1* and therefore attenuating microgliosis. Gender differences were hypothesized to be due to a sex-determining region Y element in the CHGB promoter that might act as a suppressor of transcription in males [94]. Of note, overexpression of the more common allelic variant CHGBP413 in SOD1 G37R mice significantly accelerated disease progression and pathological changes, similarly to the ALS course described in women of Japanese and French/Canadian origins carrying CHGBP413L allele, who presented an earlier ALS disease onset [94]. Moreover, peroxisome proliferator-activated receptor-γ Coactivator (PGC) -1α, a transcriptional co-activator that regulates the cellular response to metabolic demands, has been described as a disease modifier of human and experimental ALS, with a sex-dependent effect [95]. In ALS-transgenic mice, deficiency of PGC-1α accelerated disease onset and had a borderline survival effect only in the male group [95]. This modulating effect of PGC-1α on age of onset and survival was also revealed in two large ALS populations, confirming that it acts in a male-specific manner [95]. In contrast to evidence of these disease-modifying effects of PGC-1α in males, Kaneb et al. [96] reported an unexpected dose-dependent negative effect of metformin, an inductor of the production of PGC-1α, on the onset and disease progression in female SOD1 G93A transgenic mice, and this harmful effect of metformin only in female mice has been hypothesized to be due to inhibition of 17β-estradiol production caused by metformin itself [97].

Sex differences in disease phenotype have also been explored in light of the effects on mitochondrial function of mutant SOD1 in male and female ALS transgenic mice and in cellular models overexpressing SOD1 with the G93A mutation [98]. In particular, in SOD1 G93A transgenic mice, mutant SOD1 has been shown to be accumulated in the mitochondrial intermembrane space (IMS), thus determining mitochondrial dysfunction. A distinct mitochondrial unfolded protein response (UPR^mt^), induced by misfolded proteins in the IMS and mediated by the estrogen receptor alpha (ERα), was found to upregulate the proteasome and the IMS serine protease Omi (also known as high-temperature requirement factor A2 -HtrA2) activity [99]. A higher activity of these two mitochondrial pathways was detected in female SOD1G39A mice, but not in males, highlighting a significant sex difference in the IMS-UPR^mt^ [99]. Furthermore, this increased response was not observed in the absence of ERα, suggesting that sex differences in the disease phenotype could be linked to differential activation of the ERα axis of the IMS-UPR^mt^ [99].

More recently, studies on animal models have also been helpful in exploring, together with the motor phenotype, behavior differences between the two sexes: SOD1 G93A males have been shown to display reduced locomotion and exploration and increased anxiety-like behaviors, as well as an increased conditioned cue-freezing response, while impaired intermediate-term spatial memory may be reported in SOD1 G93A females [100].

Crucially, taking into account the sexual dimorphism hypothesized to be involved in pathogenic mechanisms underlying ALS, gender has also emerged as a possible factor influencing response to treatment. As an example, Torres et al. [101] studied the effect of a dietary supplementation of Docosahexaenoic acid (DHA), an essential fatty acid modulating key nervous system functions, including neuroinflammation, in SOD1 G93A mice. The Kaplan–Meyer survival analysis revealed a longer survival of G93A male mice under DHA-supplemented diet, while female G93A mice survival did not exhibit any differences. In the same way, weight loss, indicating motor neuron impairment in this model, was significantly influenced by diet in G93A male mice, but not in females [101]. On the contrary, anti-inflammatory indexes were equally affected in males and females, suggesting that only males, in addition to changes in fatty acid profiles, are benefited by yet-unknown mechanisms dependent on dietary DHA content [101]. Moreover, a previous study by Cacabelos et al. [102] has shown that high content of unsaturated fatty acids in the diet may have a negative effect on survival of SOD1 G93A female mice, causing an increased production of mitochondrial free radical species. In addition, Bame et al. [103] demonstrated that the beneficial effect of a dietary supplementation of methionine sulfoximine on the survival of SOD1 G39A mice, mostly observed in females, was completely abolished by ovariectomization or castration, suggesting that the therapeutic effect of this supplementation may involve enzymes and/or pathways influenced by sex hormones [103].

**Figure 2:**
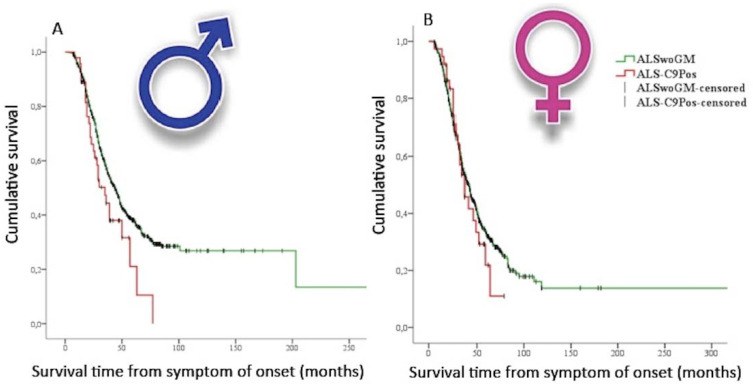
Kaplan–Meier plots of survival probabilities, stratifying the studied sample by sex [10]: shorter survival time is displayed in amyotrophic lateral sclerosis (ALS) patients carrying C9orf72 repeat expansion (ALS-C9Pos) (red line) compared to ALS patients without documented genetic mutations (ALSwoGM) (green line) only for males. (**A)**: (male): Log-rank χ2 = 4.33, *p* = 0.037; median survival was 35 months (95% CI. 26–44) for ALS-C9Pos (*n* = 47) and 44 months (95% CI. 40–48) for ALSwoGM (*n* = 681). (**B**): (female): Log-rank χ2 = 0.43, *p* = 0.510; median survival was 37 months (95% CI. 26–47) for ALS-C9Pos (*n* = 37) and 42 months (95% CI. 37–46) for ALSwoGM (*n* = 559). +: censored cases. (Derived and modified from Trojsi et al. [10], open access article distributed under the terms and conditions of the Creative Commons Attribution (CC BY) license (http://creativecommons.org/licenses/by/4.0/)).

In humans, most population-based or cohorts studies mainly focused on the effects of sex–genetics interplay in patients carrying *C9orf72* repeat expansion. Remarkably, Rooney et al. [9] performed an analysis of the prognostic characteristics of the *C9orf72* repeat expansion in 4925 ALS cases from Dutch, Irish, and Italian population-based national registers and from two (Belgian and UK) clinical research center cohorts. In this study, the authors revealed a reduced survival rate in male spinal onset patients carrying *C9orf72* repeat expansion when compared to females with the same type of onset. Moreover, this group of patients showed a shorter diagnostic delay, suggesting a rapidly progressing disease. These results were in line with those from Trojsi et al. [10], who performed a multicenter case-control study on data collected from 1417 Italian patients, stratified on the basis of the genetic data. In particular, the patients carrying *C9orf72* repeat expansion were younger at onset, at first clinical observation, and at diagnosis and exhibited higher odds of bulbar onset, FTD diagnosis, and family history of ALS, FTD, and Alzheimer’s disease. When stratifying the ALS population by sex, only men carrying *C9orf72* repeat expansion showed a shorter survival compared to apparently SALS men (Figure 2).

With regard to evidence of potential sex–genetics interplay in *C9orf72* repeat expansion (C9+) carriers, a recent meta-analysis on sex differences in genetic mutations in ALS and FTD showed that in women there has been a higher prevalence of expanded *C9orf72*-related ALS [104]. This finding may be explained on the basis of potential sex-related factors, including environmental, lifestyle, or hormonal factors, that may moderate pathogenic mechanisms and influence an older age of onset and longer survival in women, allowing for reaching the age of complete penetrance and expression of the *C9orf72*-related ALS phenotype [23,104], considering that penetrance of *C9orf72* expansions has been shown to increase with age [23,27]. Conversely, men carrying *C9orf72* expansion have been recognized to be more prone to develop ALS at a younger age [105]. Current explanations for higher male prevalence of SALS include their greater exposure to environmental risk factors (e.g., pesticides, greater amounts of physical activity, and occupations in the armed forces) [106,107], and there is no reason to assume that men with *C9orf72*-related ALS may have less exposure to these risk factors [104]. Compounding this sex difference in life expectancy, men with *C9orf72* mutations probably may have a shorter disease course [108].

Other studies did not confirm the potential interaction between sex and occurrence of *C9orf72* repeat expansion. In a population-based study by Chiò et al. [6], genetic mutations did not modify the effect of sex on clinical phenotype, neither motor nor cognitive. Similarly, Glasmacher et al. [109] did not find an association between sex and survival in *C9orf72* repeat expansion carriers.

## 5. Conclusions and Future Perspectives

ALS is a complex disease, and this complexity is mostly due to the heterogeneity of its phenotype, influenced by genetics through oligogenic mechanisms, that is, through gene–gene (or protein–protein) interactions [12,19] and by demographic factors, including age and sex, and by environment, which have longstanding negative effects, probably interacting with genetics through epigenetic mechanisms (i.e., interactomics) [38,47,110]. It is important that this complex scenario is taken into account when exploring the multiple disease pathways involved in ALS and when approaching therapy development. As personalized healthcare is taking hold in the common medical procedures, sex-specific differences, together with omics approaches (i.e., genomics, proteomics, metabolomics, epigenomics, and interactomics) have to be taken into account to shed further light on unresolved genetic causes, postgenomic effects, and molecular pathways involved in ALS and associated therapeutic strategies in the perspective of optimizing a precision medicine approach.

Most differences between the two sexes in the pathogenic mechanisms triggering ALS have been reported in preclinical models, mainly investigating disease pathways in SOD1G93A transgenic mice. Future research should explore potential sex differences in the ALS-related pathogenic mechanisms, such as, with regard to *C9orf72*-related ALS, gain of neurotoxic protein (RNA or dipeptide repeat) aggregates. Moreover, longitudinal incidence studies could be a relevant manner through which sex differences in survival and disease course may be accounted for when examining genetically well-characterized ALS populations, especially for *C9orf72*-related ALS.

## Figures and Tables

**Table 1:** Research studies analyzing the role of sex–genetics interplay in the pathogenesis and clinical phenotype of ALS.

Ref.	Authors (date)	Methods	Findings
[92]	Hayes-Punzo et al. (2012)	preclinical study in SOD1G93A rats	no significant effect of gonadectomy on disease onset and progression in both sexes
[96]	Kaneb et al. (2011)	preclinical study in SOD1G93A mice	metformin is unable to reduce pathology at any dose and has a dose-dependent negative effect on the clinical phenotype in female mice
[91]	Li et al. (2012)	preclinical study in SOD1G93A rat neural progenitor cells (rNPCs)	SOD1G93A overexpression significantly reduces cell proliferation in male cells but not female cells
[103]	Bame et al. (2012)	preclinical study in SOD1G93A mice	methionine sulfoximine treatment improves the survival of both male and female mice, but the effects are significantly greater on female mice; this effect is absent after ovariectomy or castration
[95]	Eschbach et al. (2013)	clinical study of a German ALS cohort (*n* = 590 (237F, 353M)) and of an independent Swedish ALS (confirmation) cohort (*n* = 464 (196F, 268M)) preclinical study in SOD1G93A mice	deficiency in the co-activator PGC-1α, a regulator of the cellular response to metabolic demands, may influence age of onset and survival in a male-specific manner
[102]	Cacabelos et al. (2014)	preclinical study in SOD1G93A mice	both survival and clinical evolution are dependent on dietary fatty acid unsaturation and gender, with high unsaturated diet leading to loss of the disease-sparing effect of the feminine gender
[98]	Cacabelos et al. (2016)	preclinical study in SOD1G93A mice Neuro-2A cells expressing G93A mutant or non-mutated human SOD1	ALS-associated SOD1 mutation leads to delayed mitochondrial dysfunction in female mice in comparison with males; overexpression of SOD1G93A in Neuro-2A cells reduced complex I function, and this loss of function is prevented by 17β-estradiol pretreatment
[94]	Ohta et al. (2016)	preclinical study in SOD1G37R mice and double transgenic mice overexpressing CHGB species and mutant SOD1G37R; Neuro-2A cells expressing human chromogranin B (CHGB) P413L; clinical study of Japanese (*n* = 141), French/Canadian (n = 289), French (n = 527), and in Swedish (n = 453) cohorts	the expression of CHGB 413L allelic variant in SOD1G37R mice is related to pathological changes and earlier ALS onset, specifically in female mice; in humans, the sex-related effects of CHGB variants on ALS onset are still debated
[99]	Riar et al. (2017)	preclinical study in SOD1G93A mice and Estrogen Receptor α (ERα)-knockout mice with the G93A-SOD1 mutation (ERaKO-G93A)	sex differences in the disease phenotype could be linked to differential activation of the mitochondrial intermembrane space mitochondrial unfolded protein response (IMS-UPR^mt^), probably related to ERα axis
[93]	Yan et al. (2018)	preclinical study in SOD1G93A mice	ovariectomy is associated with earlier ALS onset and attenuated the anti-inflammatory and anti-apoptotic actions of estrogen in SOD1G93A transgenic mice
[105]	Williams et al. (2013)	clinical study of Australian cohorts (FALS = 193; SALS = 559; HCs = 170)	gender-specific differences for age of onset in *C9orf72*-linked ALS: male subjects are more likely to express the disease at a younger age
[9]	Rooney et al. (2017)	case-control, population-based/multicenter study (*n* = 5106 (2053F, 2872M) ALS patients)	interaction between gender and *C9orf72* repeat expansions may have negative prognostic implications in men with spinal onset disease
[10]	Trojsi et al. (2019)	case-control, multicenter study (*n* = 1324 (596F, 728M) ALS patients)	carrying the *C9orf72* repeat expansion is an independent factor negatively impacting survival time in men but not in women
